# Detection and Classification of Immature Leukocytes for Diagnosis of Acute Myeloid Leukemia Using Random Forest Algorithm

**DOI:** 10.3390/bioengineering7040120

**Published:** 2020-10-01

**Authors:** Satvik Dasariraju, Marc Huo, Serena McCalla

**Affiliations:** 1 iResearch Institute, Glen Cove, NY 11542, USA; marchuo@stanford.edu (M.H.); drmccalla@iresearchinstitute.com (S.M.); 2 The Lawrenceville School, Lawrenceville, NJ 08648, USA; 3 School of Engineering, Stanford University, Stanford, CA 94305, USA

**Keywords:** acute myeloid leukemia, peripheral blood smear, immature leukocyte, segmentation, cytomorphology, machine learning, random forest, feature importance, computer-aided diagnosis

## Abstract

Acute myeloid leukemia (AML) is a fatal blood cancer that progresses rapidly and hinders the function of blood cells and the immune system. The current AML diagnostic method, a manual examination of the peripheral blood smear, is time consuming, labor intensive, and suffers from considerable inter-observer variation. Herein, a machine learning model to detect and classify immature leukocytes for efficient diagnosis of AML is presented. Images of leukocytes in AML patients and healthy controls were obtained from a publicly available dataset in The Cancer Imaging Archive. Image format conversion, multi-Otsu thresholding, and morphological operations were used for segmentation of the nucleus and cytoplasm. From each image, 16 features were extracted, two of which are new nucleus color features proposed in this study. A random forest algorithm was trained for the detection and classification of immature leukocytes. The model achieved 92.99% accuracy for detection and 93.45% accuracy for classification of immature leukocytes into four types. Precision values for each class were above 65%, which is an improvement on the current state of art. Based on Gini importance, the nucleus to cytoplasm area ratio was a discriminative feature for both detection and classification, while the two proposed features were shown to be significant for classification. The proposed model can be used as a support tool for the diagnosis of AML, and the features calculated to be most important serve as a baseline for future research.

## 1. Introduction

Acute myeloid leukemia (AML) is the deadliest of the four types of leukemia, accounting for 11,000 annual deaths in the US with an average five-year survival rate of 28.7% [1]. AML is characterized by the overproduction and accumulation of immature leukocytes, specifically myeloid precursors, in the bone marrow and peripheral blood. The immature white blood cells prevent the functions of the bone marrow, including the production of red blood cells and platelets, which makes the immune system vulnerable [2,3]. Detecting and classifying immature leukocytes is crucial for the diagnosis of AML.

Progressing rapidly, AML can be fatal within months or even weeks if not diagnosed and treated immediately [4]. Hence, accurate and quick diagnosis is necessary for AML patients. Microscopic examination of peripheral blood smears is the standard procedure for the diagnosis of leukemia, but other procedures are also used [5]. Manual blood smear examination is labor intensive and time consuming [6]. Moreover, manual examination is prone to considerable inter- and intra-observer variation of standards, as well as biases such as tiredness and operator experience [7]. Depending on the experience of the hematologist, manual examination has an error rate of 30% to 40% [8].

In developing countries such as Nicaragua, diagnosis takes 29 days to be reached due to lack of access to healthcare and physician delay [9,10,11]. The current method of diagnosis is unsatisfactory and a quick, accurate method is required. An automated approach will enable standardized and efficient screening for immature leukocytes, thus overcoming the limitations of the current manual method for diagnosis, especially in developing countries. Since different types of leukocytes vary in cytomorphology, detection and classification of immature leukocytes can be formulated as a machine learning classification task based on morphological features [12]. Previous studies on the computer-aided detection of leukemia have mainly focused on acute lymphoblastic leukemia (ALL) [12]. Abdeldaim et al. [13] used the ALL Image Database (ALL-IDB) [14] to train a k-nearest neighbors (k-NN) classifier with 95.99% accuracy for classification of ALL subtypes. Classification performance was improved by Shafique and Tehsin [15], who applied a convolutional neural network (CNN) on images from the ALL-IDB repository and achieved 99.50% accuracy for detection of ALL with 96.74% accuracy for classification. Research aiming to detect and classify AML has obtained lower performance compared to studies on ALL due to the high diversity in cytomorphology of AML cells [12].

A multitude of segmentation techniques, morphological features, and machine learning classifiers have been employed in the literature. Kazemi et al. [16] segmented 165 images of four subtypes of AML cells with k-means clustering. Using a support vector machine (SVM), 95% accuracy was obtained for detection of AML cells and 87% accuracy was obtained for classification into four of the eight French-American-British (FAB) subtypes of AML. The study utilized 60 cytomorphological features for classification, yet the most important features were not established. E.S. Wiharto et al. [17] selected three morphological features and calculated the importance of each feature. Evidently, there is a lack of standardization in the number and type of features used for selection, which needs to be addressed. Classification into two FAB subtypes was obtained with 67.28% accuracy [17]. Harjoko et al. [18] used active contour segmentation, extracted six morphological features, and used the momentum backpropagation neural network to classify three subtypes of AML with 93.57% accuracy. Despite the high accuracy, the proposed model was limited by precision and sensitivity values below 85%. W. Wiharto et al. [19] classified three immature leukocytes in AML cells from a small dataset of 50 images. After segmentation through Otsu thresholding, which is a common method used in literature, three morphological characteristics were extracted and ranked based on importance for classification. To overcome the imbalance of data, synthetic minority oversampling technique (SMOTE) was employed with a random forest algorithm, which obtained 90% accuracy. The research displayed that imbalanced data, which has limited many previous models, can be overcome through selection and tuning of a random forest classifier. Matek et al. [12] assembled an image dataset of 18,365 leukocytes [20,21] and employed a CNN for classification. For binary classification between immature and mature blood cells, the CNN obtained an area under curve (AUC) of the receiver operating characteristic (ROC) of 0.992, which is the current state of art. Despite high performance in detection of immature leukocytes, the CNN achieved precision of below 65% for the majority of immature leukocyte classes, which was attributed to the imbalance of data across different classes. Overall, previous studies are limited by the use of small data sets, which may lead to overfitting, and imbalance across classes. In addition, given the importance of feature selection in machine learning classifiers, the lack of uniformity in the type of features used for classification of AML cells still needs to be addressed by identifying the most important features. 

The purpose of this research is to develop a model capable of accurately detecting and classifying immature leukocytes in AML cells from an imbalanced dataset into four types (erythroblasts, monoblasts, promyelocytes, and myeloblasts) with a random forest algorithm. Detection and classification of immature leukocytes will greatly aid the clinical diagnosis of AML. To add to the limited set of color features used for classification of leukocytes [16], two new features for classification of leukocytes, specifically the average and standard deviation of nucleus color intensity in the B channel of LAB color space, are proposed and demonstrated to be discriminative. Furthermore, the most important features for both detection and classification are calculated and ranked using the Gini importance, which is defined as the loss of Gini impurity caused by each feature in the random forest. To the best of the authors’ knowledge, this is the first study that calculates the Gini importance of a multitude of morphological features for classification of leukocytes in AML.

## 2. Materials and Methods

### 2.1. Dataset

**Figure 1:**
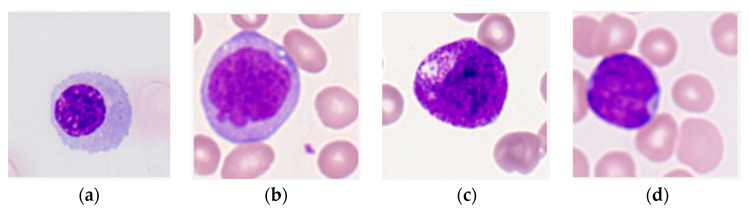
Sample images of the four types of immature leukocytes in acute myeloid leukemia (AML) patients [20,21] used in this study. Each image is centered around a leukocyte and contains background cells. (**a**) Erythroblast; (**b**) monoblast; (**c)** promyelocyte; (**d)** myeloblast.

Labelled images of leukocytes from the peripheral blood of 100 AML patients and 100 healthy controls were collected from the dataset assembled by Matek et al. [20] in The Cancer Imaging Archive [21]. The dataset contains a total of 18,365 images centered around a leukocyte with ground truth labels that classify images by leukocyte type (Figure 1). Ground truth annotations were made by a medical examiner experienced in cytomorphology [12,20].

Table 1 displays the number of images from each leukocyte type used in this study. Classes of immature leukocytes with less than 20 images (bilobed promyelocytes and metamyelocytes) were omitted because after splitting into training and testing sets, an insufficient number of images would remain in the testing set for statistically significant results. For the myeloblast class, which contained over 3000 images, a random sample of 500 images were used. In total, 731 immature leukocytes were used with considerable imbalance across classes. Data augmentation was not utilized to increase samples in minority classes because morphological features are invariant regardless of rotations and reflections. A total of 600 mature leukocytes were used to provide a control group for the detection of mature leukocytes.

### 2.2. Methodology

The methodology consisted of four main phases: segmentation, feature extraction, classification, and calculation of feature importance. During segmentation, binary masks of the cell and nucleus were obtained for each image. A total of 16 features were extracted to be inputted into a random forest algorithm for classification between immature and mature cells, as well as further classification of immature cells. Finally, the importance of each feature was calculated using the metrics of the random forest algorithm. The project was coded in the Python programming language [22] with numerous open source libraries [23,24,25,26,27], including sci-kit image for feature calculation and sci-kit learn for machine learning implementation.

#### 2.2.1. Segmentation

**Figure 2:**
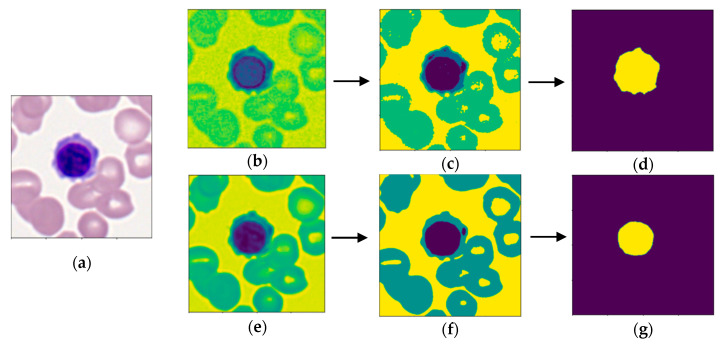
Sample of segmentation process. Images (**b**–**d**) display segmentation of the cell, while images (**e**–**g**) show segmentation of the nucleus. (**a**) Raw image of an erythroblast [20,21]; (**b**) conversion to LAB color space; (**c)** multi-Otsu thresholding; (**d)** binary mask of cell; (**e**) conversion to grayscale format; (**f**) multi-Otsu thresholding; (**g**) binary mask of nucleus.

The objective of segmentation (see Figure 2) was to obtain masks of the cell and nucleus, from which morphological features could be extracted from. To obtain a cell mask, each image was converted to LAB format to better differentiate the cytoplasm from background cells [16,28] (see Figure 2b). Multi-Otsu thresholding [29] with three thresholds was used to group image pixels into four clusters: image background, background cells, cytoplasm of cell, and nucleus of cell (see Figure 2c). Since the nucleus and cytoplasm had the highest and second-highest intensities, respectively, components below the second multi-Otsu threshold were removed. Morphological dilation followed by erosion by the same factor was used to separate noise from the region of interest (ROI). A few images contained multiple stained leukocytes, thus a positional filter was applied to only select the ROI in the center of the image. The image was smoothed with the removal of small objects with an area below 2000 to obtain the final cell mask, as displayed in Figure 2d.

A similar process was carried out to obtain a binary mask of the nucleus. Since the nucleus was always the darkest component, the image was converted to gray scale for the best discrimination of the nucleus, as shown in Figure 2e. Multi-Otsu thresholding with two thresholds was utilized to group pixels into either the background, background cells and cytoplasm, or nucleus of the cell (see Figure 2f). In some images, noise would be grouped in the same cluster as the nucleus. To overcome this, only connected components containing the center pixel were kept to discern the final binary nucleus mask. As exhibited in Figure 2g, the nucleus mask was subtracted from the cell mask to obtain a binary mask of the cytoplasm.

The proposed segmentation procedure successfully segmented 1070 out of 1274 images (83.99%). The majority of failed segmentation can be attributed to background cells overlapping with the ROI and stain obscuring the ROI. Given that the dataset was collected for a study with a CNN, which does not require segmentation and feature extraction, the segmentation results are acceptable. Table 2 displays the number of images remaining in each class after segmentation.

#### 2.2.2. Feature Extraction

The purpose of feature extraction was to obtain a set of descriptors that are discriminative for classification of leukocytes. From each image, 16 cytomorphological features were extracted, which could be divided into four categories: nucleus size, nucleus shape, elliptical features, and color features. Nucleus size features consisted of area, perimeter, area to perimeter ratio, equivalent diameter [19], and nucleus to cytoplasm area ratio (N:C ratio) [30]. Size features of the nucleus are important for classifying leukocytes because as leukocytes mature, the nucleus decreases in size [16]. The nucleus shape features include circularity (Equation (1)), solidity (Equation (2)), and compactness (Equation (3)) calculated as follows:

(1) $$circularity=\frac{4\pi A}{P^{2}}$$

(2) $$solidity=\frac{A}{A_{c}}$$

(3) $$compactness=\frac{P^{2}}{A}$$

In the above equations, A is the area of the nucleus, $A_{c}$ is the area of the convex hull of the nucleus, and P is the perimeter of the nucleus. Elliptical features included eccentricity, minor axis length, major axis length, and elongation. Eccentricity (Equation (4)) and elongation (Equation (5)) were calculated as follows:

(4) $$eccentricity=\frac{D_{f}}{l_{M}}$$

(5) $$elongation=1-\frac{l_{m}}{l_{M}}$$

here, $D_{f}$ is the focal distance, $l_{M}$ is the major axis length, and $l_{m}$ is the minor axis length. Due to the unique morphological characteristics of leukocytes, the standard features used for classification of tumors are not sufficient [28]. We propose two new color features in this study: average and standard deviation of nucleus in the B channel of LAB color space. Additionally, two cytoplasm color features conceived by Ghane et al. [28] are also used. Color features have been demonstrated in previous studies to be significant for classification of leukocytes [6,16]. All 16 features for each image were added to a feature matrix, which served as the input for the classifier. Morphological features of the whole cell were not used, with the exception of the cytoplasm area in the N:C ratio, because the positioning of background cells dictates the shape and orientation of the cytoplasm of the leukocyte. Therefore, the shape of the cytoplasm would be highly variable and not correlated with leukocyte type. While the study did not utilize texture and fractal features, previous works have utilized them and obtained successful results [16]. Future studies can employ texture and fractal features, which may improve classification performance.

#### 2.2.3. Classification

A random forest algorithm was chosen for classification because of its higher performance with imbalanced data when compared to other machine learning classifiers [31,32,33,34]. A random forest algorithm is an ensemble classifier that combines a specified number of decision trees and takes the majority decision to predict classification, thus preventing overfitting. 

In the classification step, binary classification between immature and mature leukocytes was first performed, followed by classification of immature leukocytes into four types. For binary classification, 80% of the data in the features matrix was used for training and 20% was reserved for testing of the model. For multiclass classification of immature leukocytes, 70% of the data was used for training and 30% was used as the testing set. All splitting of data into training and testing sets was randomized. A random forest classifier with 100 trees was initially tested and evaluated for both binary and multiclass classification. Binary classification was quantitatively evaluated on the testing set with accuracy, precision, recall (equivalent to sensitivity), and specificity as performance metrics. The performance metrics were based on the possible outcomes of classification: true positive (*TP*), true negative (*TN*), false positive (*FP*), and false negative (*FN*). Binary classification performance metrics, namely accuracy (Equation (6)), precision (Equation (7)), recall (Equation (8)), and specificity (Equation (9)), were defined as follows:

(6) $$accuracy=\frac{TP+TN}{TP+TN+FP+FN}$$

(7) $$precision=\frac{TP}{TP+FP}$$

(8) $$recall=\frac{TP}{TP+FN}$$

(9) $$specificity=\frac{TN}{TN+FP}$$

Multiclass classification was evaluated on the testing set with overall accuracy, precision for each class, and recall for each class. For multiclass classification, a true positive refers to an image correctly being given a label; a true negative refers to an image correctly being not given to a label; a false positive refers to an image incorrectly being given a label; and a false negative refers to an image incorrectly not being given a label. The multiclass classification model was optimized through a search of ten randomized combinations of random forest hyperparameters. Combinations of parameters were evaluated with the mean of precision scores across classes during five-fold cross validation on the training set. The class weight parameter, which is set to none in the default setting, was selected to be balanced to overcome the imbalance of data. A balanced random forest classifier uses that size of each class to assign weights inversely proportional to the frequency of each class. The optimized model was assessed with the same metrics as the initial multiclass classifier. 

#### 2.2.4. Calculation of Feature Importance

The importance of each feature was quantitatively evaluated with Gini importance, also called mean decrease in impurity (MDI). The Gini importance of a feature in a random forest algorithm is defined as the mean reduction in Gini impurity across all decision trees caused by the feature [34]. For each of the 16 features, the five most important features were ranked to establish which features are most crucial for classifying leukocytes. 

## 3. Results and Discussion

### 3.1. Detection of Immature Leukocytes

After the random forest model was trained, the performance was evaluated with the previously listed performance metrics. Table 3 displays the performance of the model for binary classification between immature and mature leukocytes on the training and testing set with the random forest algorithm. The model classified all the images in the training set correctly, with 92.99% accuracy on the testing set. Precision, recall, and specificity values for the testing set also were above 90%. Table 4 displays the confusion matrix for binary classification, which is the number of correct and incorrect predictions for each class. 

**Figure 3:**
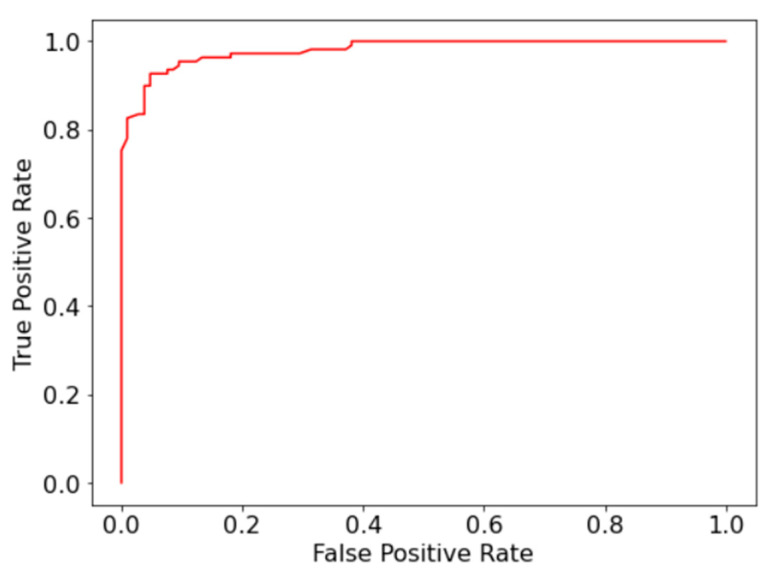
Receiver operating characteristic (ROC) curve for binary classification between immature and mature leukocytes.

Compared to the accuracy and recall of the model, the precision and specificity are slightly lower due to the number of false positives. Although not ideal, high recall is preferred over precision for fatal diseases such as AML. Figure 3 displays the receiver operating characteristic (ROC) curve, which plots the false positive rate against the true positive rate for the binary classifier.

The area under the curve of the ROC curve (AUC-ROC) is 0.98, which is comparable to the current state of art model in the study by Matek et al. [12], which achieved an AUC-ROC of 0.992. The high-performance metrics display that the proposed random forest classifier can be used as an effective tool for identifying immature cells in the diagnosis of AML.

### 3.2. Classification of Immature Leukocytes

For multiclass classification, the initial random forest model obtained precision and recall above 85% for all classes except the promyelocyte class. The optimized model, which was constructed with average precision as the scoring metric, obtained precision above 65% for all classes (see Table 5). The model achieved above 90% precision and recall for the myeloblast class, which is the most common immature leukocyte in AML patients.

The results are superior to previous state of art [12], which achieved precision scores of below 65% for classification of most immature leukocyte types despite very high performance in detection. While past research has obtained low performance on the minority class, the proposed model achieved 100% recall on the monoblast class. The model had the lowest performance on the promyelocyte class, with precision and recall scores below 85% for both the initial and optimized model. Table 6 displays the confusion matrix for the optimized multiclass model, which shows that the majority of incorrect predictions on the promyelocyte class labeled the image as a myeloblast. 

The lower performance on the promyelocyte class can be attributed to the fact that promyelocytes and myeloblasts are consecutive steps in the cell lineage of myeloid cells, therefore the two classes share morphological characteristics [35]. Likewise, the majority of incorrect predictions on the myeloblast class classified the image as a promyelocyte due to the similarity between classes.

### 3.3. Most Important Features

Based on Gini importance, the most important features for detection (see Table 7) and classification (see Table 8) were calculated. The five most important features for detection are all either nucleus size features or elliptical features, which is explained by the trait of leukocyte to decrease in size as the cell matures [16].

The N:C ratio was calculated to be a significant discriminator for both detection and classification of immature leukocytes. This finding is supported by previous research that classified hemocyte precursors [6]. For classification, the Gini importance of the two proposed nucleus color features were the highest of all 16 features, while cytoplasm color features from [28] were also displayed to be discriminative.

## 4. Conclusions

To overcome the limitations of the manual diagnosis methodology for AML, a random forest model for automatic detection and classification of immature leukocytes was presented. The model was capable of detecting immature leukocytes with 93% accuracy and 0.98 AUC-ROC, which is on par with the current state of art [12]. Furthermore, the model achieved precision of above 65% for each of the four immature leukocyte classes during multiclass classification, despite imbalance in numbers across classes, which is an improvement over previous research. Using Gini importance, N:C ratio was determined to be significant for both detection and classification, while the proposed color features of the nucleus in the B channel of LAB color space were calculated to be important for classification.

Applications of the study are two-fold. While the proposed model cannot diagnose AML alone, it can be used as an effective support tool for doctors to reduce the time and cost required for the diagnosis of AML. The high accuracy of the model in binary classification demonstrates that the model can serve as an efficient screening tool, which can rapidly identify potentially cancerous cells for further examination by a doctor [36,37,38]. The proposed model can expedite the detection of AML by identifying immature leukocytes, especially in developing countries where diagnosis takes numerous weeks, and potentially save lives because early diagnosis is vital for treatment success in AML patients [9,39]. In addition, the precise classification of immature leukocytes can aid in treatment and prognosis decisions, which differ based on the type of cancerous cell [40,41]. The second application of this study is in future research, where the features calculated to be most important and the proposed features can be used to elevate the classification performance.

An important future direction is to gather a comprehensive dataset and develop a machine learning classifier that can classify all the types of immature leukocytes and work with imbalanced data. Future studies can expand on this work by calculating and ranking the importance of additional morphological features for the classification of leukocytes. Improving the discrimination between similar cell types, such as myeloblasts and promyelocytes, is also an avenue for future work. The difficulty of differentiating myeloblasts and promyelocytes can potentially be overcome by identifying features that are especially discriminative for the two cell types and training a specialized model to discriminate between the two cell types. Research on leukemia detection has obtained very promising results, and further work is required to develop systems that can be completely integrated into the clinical diagnosis method. Contributions of this study are an accurate model for detecting and classifying immature leukocytes, as well as calculation of the most important morphological features, which provide a basis for future research on computer-aided diagnosis of leukemia.

## Figures and Tables

**Table 1:** Data coverage for four immature leukocyte types, total immature leukocytes, and total mature leukocytes.

Immature Cells				Mature Cells	Total
Erythroblasts	Monoblasts	Promyelocytes	Myeloblasts		
78	26	70	500	600	1274

**Table 2:** Amount of data remaining after segmentation for four immature leukocyte types, total immature leukocytes, and total mature leukocytes.

Immature Cells				Mature Cells	Total
Erythroblasts	Monoblasts	Promyelocytes	Myeloblasts		
66	22	40	419	457	1070

**Table 3:** Performance metrics of the optimized model for binary classification between immature and mature leukocytes on training and testing sets.

Set	Accuracy	Precision	Recall (Sensitivity)	Specificity
Training Set	100%	100%	100%	100%
Testing Set	92.99%	91.23%	95.41%	90.48%

**Table 4:** Confusion matrix of the optimized model for binary classification on testing set.

	Predicted Label	
	Mature Leukocyte	Immature Leukocyte
Mature Leukocyte	95	10
Immature Leukocyte	5	104

**Table 5:** Performance metrics for the initial and optimized random forest classifiers on each of the four immature leukocyte types.

Model	Class	Precision	Recall	Overall Accuracy
Initial Random Forest	Erythroblast	100%	91.30%	93.45%
	Monoblast	87.50%	100%	
	Promyelocyte	62.50%	83.33%	
	Myeloblast	96.75%	94.44%	
Optimized Random Forest	Erythroblast	100%	91.30%	93.45%
	Monoblast	77.78%	100%	
	Promyelocyte	69.23%	75%	
	Myeloblast	97.56%	96.77%	

**Table 6:** Confusion matrix for optimized classification of immature leukocytes.

	Predicted Label			
	Erythroblast	Monoblast	Promyelocyte	Myeloblast
Erythroblast	21	0	0	2
Monoblast	0	7	0	0
Promyelocyte	0	0	9	3
Myeloblast	0	2	4	120

**Table 7:** The five most important features for detection of immature leukocytes based on Gini importance.

Feature	Gini Importance
N:C Ratio	0.2801
Area to Perimeter Ratio	0.1076
Nucleus Minor Axis Length	0.0829
Nucleus Major Axis Length	0.0803
Area	0.0627

**Table 8:** The five most important features for classification of immature leukocytes based on Gini importance. Color intensity is calculated in the B channel of the component in LAB color space. Asterisk (*) indicates new features proposed in this study.

Feature	Gini Importance
Average Nucleus Color Intensity in B Channel *	0.2532
Standard Deviation of Nucleus Color Intensity in B Channel *	0.1853
N:C Ratio	0.1765
Standard Deviation of Cytoplasm Color Intensity in B Channel	0.0618
Average Cytoplasm Color Intensity in B Channel	0.0571

## References

[1] Acute Myeloid Leukemia—Cancer Stat Facts. *SEER*. https://seer.cancer.gov/statfacts/html/amyl.html.

[2] Saultz J.N., Garzon R. (2016). Acute Myeloid Leukemia: A Concise Review. J. Clin. Med..

[3] American Society of Hematology. https://www.hematology.org:443/.

[4] Kumar C.C. (2011). Genetic Abnormalities and Challenges in the Treatment of Acute Myeloid Leukemia. Genes Cancer.

[5] Ahmed N., Yigit A., Isik Z., Alpkocak A. (2019). Identification of Leukemia Subtypes from Microscopic Images Using Convolutional Neural Network. Diagnostics.

[6] Prinyakupt J., Pluempitiwiriyawej C. (2015). Segmentation of white blood cells and comparison of cell morphology by linear and naïve Bayes classifiers. Biomed. Eng. Online.

[7] Sasada K., Yamamoto N., Masuda H., Tanaka Y., Ishihara A., Takamatsu Y., Yatomi Y., Katsuda W., Sato I., Matsui H. (2018). Inter-observer variance and the need for standardization in the morphological classification of myelodysplastic syndrome. Leuk. Res..

[8] Amin M.M., Kermani S., Talebi A., Oghli M.G. (2015). Recognition of Acute Lymphoblastic Leukemia Cells in Microscopic Images Using K-Means Clustering and Support Vector Machine Classifier. J. Med. Signals Sens..

[9] De Angelis C., Pacheco C., Lucchini G., Arguello M., Conter V., Flores A., Biondi A., Masera G., Baez F. (2012). The Experience in Nicaragua: Childhood Leukemia in Low Income Countries—The Main Cause of Late Diagnosis May Be ‘Medical Delay. Int. J. Pediatr..

[10] Salah H.T., Muhsen I.N., Salama M.E., Owaidah T., Hashmi S.K. (2019). Machine learning applications in the diagnosis of leukemia: Current trends and future directions. Int. J. Lab. Hematol..

[11] Howell D.A., Smith A.G., Jack A., Patmore R., Macleod U., Mironska E., Roman E. (2013). Time-to-diagnosis and symptoms of myeloma, lymphomas and leukaemias: A report from the Haematological Malignancy Research Network. BMC Blood Disord..

[12] Matek C., Schwarz S., Spiekermann K., Marr C. (2019). Human-level recognition of blast cells in acute myeloid leukaemia with convolutional neural networks. Nat. Mach. Intell..

[13] Abdeldaim A.M., Sahlol A.T., Elhoseny M., Hassanien A.E. (2018). Computer-Aided Acute Lymphoblastic Leukemia Diagnosis System Based on Image Analysis. Advances in Soft Computing and Machine Learning in Image Processing.

[14] Labati R.D., Piuri V., Scotti F. All-IDB: The acute lymphoblastic leukemia image database for image processing. Proceedings of the 2011 18th IEEE International Conference on Image Processing.

[15] Shafique S., Tehsin S. (2018). Acute Lymphoblastic Leukemia Detection and Classification of Its Subtypes Using Pretrained Deep Convolutional Neural Networks. Technol. Cancer Res. Treat..

[16] Kazemi F., Najafabadi T.A., Araabi B.N. (2016). Automatic Recognition of Acute Myelogenous Leukemia in Blood Microscopic Images Using K-means Clustering and Support Vector Machine. J. Med. Signals Sens..

[17] Wiharto E.S., Palgunadi S., Putra Y.R. Cells identification of acute myeloid leukemia AML M0 and AML M1 using K-nearest neighbour based on morphological images. Proceedings of the 2017 International Conference on Data and Software Engineering (ICoDSE).

[18] Harjoko A., Ratnaningsih T., Suryani E., Palgunadi S., Prakisya N.P.T. (2018). Classification of acute myeloid leukemia subtypes M1, M2 and M3 using active contour without edge segmentation and momentum backpropagation artificial neural network. MATEC Web Conf..

[19] Wiharto W., Suryani E., Putra Y.R. (2019). Classification of blast cell type on acute myeloid leukemia (AML) based on image morphology of white blood cells. TELKOMNIKA (Telecommun. Comput. Electron. Control).

[20] Matek C., Schwarz S., Marr C., Spiekermann K. (2019). A Single-cell Morphological Dataset of Leukocytes from AML Patients and Non-Malignant Controls [Data set].

[21] Clark K., Vendt B., Smith K., Freymann J., Kirby J., Koppel P., Moore S., Phillips S., Maffitt D., Pringle M. (2013). The Cancer Imaging Archive (TCIA): Maintaining and operating a public information repository. J. Digit. Imaging.

[22] Van der Walt S., Colbert S.C., Varoquaux G. (2011). The NumPy Array: A Structure for Efficient Numerical Computation. Comput. Sci. Eng..

[23] The Python Language Reference—Python 3.8.5 Documentation. https://docs.python.org/3/reference/.

[24] Hunter J.D. (2007). Matplotlib: A 2D Graphics Environment. Comput. Sci. Eng..

[25] McKinney W. Data Structures for Statistical Computing in Python. Proceedings of the 9th Python in Science Conference.

[26] Van der Walt S., Schönberger J.L., Nunez-Iglesias J., Boulogne F., Warner J.D., Yager N., Gouillart E., Yu T. (2014). scikit-image: Image processing in Python. PeerJ.

[27] Pedregosa F., Varoquaux G., Gramfort A., Michel V., Thirion B., Grisel O., Blondel M., Prettenhofer P., Weiss R., Dubourg V. (2011). Scikit-learn: Machine Learning in Python. J. Mach. Learn. Res..

[28] Ghane N., Vard A., Talebi A., Nematollahy P. (2019). Classification of chronic myeloid leukemia cell subtypes based on microscopic image analysis. EXCLI J..

[29] Liao P.S., Chen T.S., Chung P.-C. (2001). A fast algorithm for multilevel thresholding. J. Inf. Sci. Eng..

[30] Mathur A., Tripathi A.S., Kuse M. (2013). Scalable system for classification of white blood cells from Leishman stained blood stain images. J. Pathol. Inform..

[31] Breiman L. (2001). Random Forests. Mach. Learn..

[32] Parmar A., Katariya R., Patel V. A Review on Random Forest: An Ensemble Classifier. Proceedings of the International Conference on Intelligent Data Communication Technologies and Internet of Things (ICICI) 2018.

[33] Khalilia M., Chakraborty S., Popescu M. (2011). Predicting disease risks from highly imbalanced data using random forest. BMC Med. Inform. Decis. Mak..

[34] Louppe G., Wehenkel L., Sutera A., Geurts P. Understanding variable importances in forests of randomized trees. Proceedings of the 26th International Conference on Neural Information Processing Systems—Volume 1.

[35] Carmona-Rivera C., Kaplan M.J., Bradshaw R.A., Stahl P.D. (2016). Neutrophil Biology. Encyclopedia of Cell Biology.

[36] Bigorra L., Merino A., Alférez S., Rodellar J. (2016). Feature Analysis and Automatic Identification of Leukemic Lineage Blast Cells and Reactive Lymphoid Cells from Peripheral Blood Cell Images. J. Clin. Lab. Anal..

[37] Khobragade S., Mor D.D., Patil C.Y. Detection of leukemia in microscopic white blood cell images. Proceedings of the 2015 International Conference on Information Processing (ICIP).

[38] Patel N., Mishra A. (2015). Automated Leukaemia Detection Using Microscopic Images. Procedia Comput. Sci..

[39] Thanh T.T.P., Vununu C., Atoev S., Lee S.-H., Kwon K.-R. (2018). Leukemia Blood Cell Image Classification Using Convolutional Neural Network. Int. J. Comput. Theory Eng..

[40] Vakiti A., Mewawalla P. (2020). Cancer, Acute Myeloid Leukemia (AML, Erythroid Leukemia, Myelodysplasia-Related Leukemia, BCR-ABL Chronic Leukemia). StatPearls.

[41] Shafique S., Tehsin S. (2018). Computer-Aided Diagnosis of Acute Lymphoblastic Leukaemia. Comput. Math. Methods Med..

